# Assessing variability in results in systematic reviews of diagnostic studies

**DOI:** 10.1186/s12874-016-0108-4

**Published:** 2016-01-15

**Authors:** Christiana A. Naaktgeboren, Eleanor A. Ochodo, Wynanda A. Van Enst, Joris A. H. de Groot, Lotty Hooft, Mariska M. G. Leeflang, Patrick M. Bossuyt, Karel G. M. Moons, Johannes B. Reitsma

**Affiliations:** Julius Center for Health Sciences and Primary Care, University Medical Center, Universiteitsweg 100, Utrecht, 3584 CG The Netherlands; Department of Clinical Epidemiology, Biostatistics and Bioinformatics, Academic Medical Center, Amsterdam, The Netherlands; Centre for Evidence-based Health Care, Faculty of Medicine and Health Sciences, Stellenbosch University, Cape Town, South Africa; Dutch Cochrane Centre, Academic Medical Center, Amsterdam, The Netherlands

**Keywords:** Meta-analysis, Diagnostic techniques and procedures/standards, Sensitivity and specificity, Data interpretation, Statistical, Bias (epidemiology)

## Abstract

**Background:**

To describe approaches used in systematic reviews of diagnostic test accuracy studies for assessing variability in estimates of accuracy between studies and to provide guidance in this area.

**Methods:**

Meta-analyses of diagnostic test accuracy studies published between May and September 2012 were systematically identified. Information on how the variability in results was investigated was extracted.

**Results:**

Of the 53 meta-analyses included in the review, most (*n*=48; 91 %) presented variability in diagnostic accuracy estimates visually either through forest plots or ROC plots and the majority (*n*=40; 75 %) presented a test or statistical measure for the variability. Twenty-eight reviews (53 %) tested for variability beyond chance using Cochran’s Q test and 31 (58 %) reviews quantified it with I^2^. 7 reviews (13 %) presented between-study variance estimates (τ^2^) from random effects models and 3 of these presented a prediction interval or ellipse to facilitate interpretation. Half of all the meta-analyses specified what was considered a significant amount of variability (*n*=24; 49 %).

**Conclusions:**

Approaches to assessing variability in estimates of accuracy varied widely between diagnostic test accuracy reviews and there is room for improvement. We provide initial guidance, complemented by an overview of the currently available approaches.

**Electronic supplementary material:**

The online version of this article (doi:10.1186/s12874-016-0108-4) contains supplementary material, which is available to authorized users.

## Background

Over the past decade, there has been a sharp increase in the number of meta-analyses of diagnostic studies published and the methods for performing such a meta-analysis have rapidly evolved [1, 2]. Analyzing the variability in results from primary studies is challenging in any type of systematic review, but it is even more difficult in systematic reviews of diagnostic studies. This is because the interest is often in two correlated estimates from the same study: pairs of sensitivity and specificity. How the variability in the results of diagnostic studies can best be assessed demands further attention.

Estimates of test accuracy are likely to differ between studies in a meta-analysis. This is referred to as variability or heterogeneity (in the broad sense of the word) [3]. Some variability in estimates can be expected simply due to chance as a result of sampling error. Even if studies are methodologically identical and carried out in the same population, their results may differ because each study only observes a sample from the entire theoretical population. When there is more variability than expected due to chance alone, this is termed statistical heterogeneity, and is referred to by some as “true heterogeneity” or simply as heterogeneity [4–6]. When there is statistical heterogeneity, it indicates that a test’s accuracy differs between studies (this is sometimes referred to as a difference in “true effects”). Review authors may be encouraged to look into possible explanations for these differences as they may have important clinical implications [3, 5]. The more variability beyond chance there is, the more difficult it is to come to strong conclusions about the clinical implications of the findings of the meta-analysis [7].

When there is a single (univariate) measure of effect, Cochran’s Q test is often used to test for variability beyond chance and I^2^ is used to quantify this variability. Unlike reviews of interventions that focus on a single measure of effect (e.g., a risk ratio or odds ratio), reviews of diagnostic studies often meta-analyze two correlated outcomes, namely sensitivity and specificity (the proportions of diseased and non-diseased that are correctly identified). Sensitivity and specificity vary inversely with the threshold at which patients are considered diseased, leading to a negative correlation between these estimates known as the threshold effect. Thresholds can be explicit, such as specific values used in laboratory tests, or implicit, such as differences in the way that imaging tests are interpreted between studies.

In a meta-analysis of diagnostic tests, the explicit or implicit thresholds of the test under study may differ across studies, leading to varying estimates of sensitivity and specificity. It is clinically relevant to know about the variability that exists beyond what could be attributed to either chance or the threshold effect. Instead of performing two separate univariate analyses of sensitivity and specificity in which it is impossible to calculate the amount of variability that is due to the threshold effect, another approach is to focus on a single parameter, such as the diagnostic odds ratio (DOR), overall accuracy, or the Youden’s index. The Moses-Littenberg summary receiver operating characteristic curve (SROC) takes this approach by modeling the relationship between accuracy and a parameter related to the threshold, namely, the proportion with positive test results [8]. More recently, however, it has been shown that hierarchical bivariate random effects models are more appropriate and more insightful, such as the bivariate random effects model proposed by Reitsma et al., which focuses on estimating a summary point and corresponding confidence region or the Hierarchical SROC (HSROC) model, which focuses on fitting a summary receiver operating characteristic (SROC) curve [9–11]. These models are both random effects models which assume that the true effects vary with a given distribution around a mean value and estimates that distribution, as opposed to fixed effect models which assume that all studies share the same common effect. The HSROC and the bivariate model are identical when no covariates are included and parameters from one model can be used to calculate those from the other [12]. The bivariate random effects analysis estimates the amount of correlation between the two outcome measures, thereby enabling the calculation of the conditional between-study variances (i.e., the variance in specificity at a fixed value of sensitivity and vice versa) that are smaller than the between-study variances from two separate univariate analyses of sensitivity and specificity in case there is a (negative) correlation between the two outcome measures*.*

The aim of this review is to survey which methods are currently used to visualize, test, measure, interpret, and inform readers about variability in results in meta-analysis of diagnostic studies. This study is complementary to another review that we have done on how sources of heterogeneity are explored [13]. In the discussion we provide initial guidance on testing for and quantifying variability in reviews of diagnostic test accuracy.

## Methods

This study was a part of a meta-epidemiologic project on systematic reviews of diagnostic studies. The goal of this project was to investigate several methodological topics such as publication bias, small sample size effects, time lag bias, quality assessment, and how sources of variability are explored. A database containing a set of reviews of diagnostic tests was established for this project.

Systematic reviews on diagnostic tests that were indexed between May 1^st^ and September 11^th^, 2012 were identified on September 12^th^ using a systematic search in EMBASE and MEDLINE (Additional file 1). As this article is about formal (statistical) methods for assessing variability in study results, we focused on the systematic reviews containing a meta-analysis. However, we additionally examined the systematic reviews captured by this search strategy in which no meta-analysis was performed for the sole purpose of investigating whether high variability in study results was a reason for not performing a meta-analysis. A meta-analysis was defined as a review in which a summary estimate for at least one diagnostic accuracy estimator was reported or in which a summary ROC curve (SROC) was provided. Reviews on prognostic tests, individual patient data reviews, conference abstracts, or written in a language other than English were excluded.

Data extraction was performed using a standardized form by one reviewer and the extraction was checked by a second reviewer. For the overarching project, general study characteristics of interest for all parts of the project, such as the type of test under study and the number of primary studies, were extracted from the reviews. For this study, information was extracted on how the results of the meta-analyses were presented graphically, which statistical tests or measures for variability were used, how the results of variability were used to guide the analysis, and how the variability in results was mentioned in the discussion and/or conclusions.

To facilitate interpretation of our results, we have provided an explanation of the terminology, different measures and statistical tests that are used when investigating variability in univariate analyses, like Cochran’s Q test, I^2^,τ^2^, and prediction intervals in Additional file 2.

## Results

### Search results

The search resulted 1273 hits after duplicates were excluded. Title screening resulted in the elimination of 1058 articles. The full text of the remaining 89 potentially relevant articles was read to determine whether articles met the inclusion criteria. In the end, 65 reviews, of which 53 contained a meta-analysis and 12 did not, were included. Additional file 3 contains the study inclusion flow chart and Additional file 4 contains a list of the included systematic reviews.

### General characteristics of the reviews

General characteristics of the 53 reviews that contained a meta-analysis can be found in Table 1. Most meta-analyses contained a low number of studies, with a median of 14 (Interquartile range (IQR): 9.5–18.5). The majority of reviews were on imaging tests (60 %), a large percentage was on lab tests (26 %), and a few were on clinical or physical examination procedures (14 %). Over half of the meta-analyses studied the accuracy of more than one test.

**Table 1:** Characteristics of meta-analyses included in the review (*n*=53)

Characteristic	N or median	% or IQR
Median number of primary studies	14	[9.5–18.5]
Median number of patients in primary studies	87	[45–182]
Type of test tests under study		
Laboratory tests	15	28 %
Image tests	32	60 %
Clinical examination	6	11 %
Meta-analyses looking at more than one test	31	58 %
Method(s) for conducting the meta-analysis†		
Univariate analysis only	13	25 %
SROC (Moses Littenberg): linear regression D on S	24	45 %
HSROC (Rutter and Gatsonis): accuracy, scale and threshold parameter	5	9 %
Bivariate random effects model (Reitsma): random effects sens & spec	13	25 %

Of the 12 systematic reviews that did not perform a meta-analysis, eight stated that they did not do so because there was too much clinical or methodological heterogeneity between the studies. None of these 12 reviews reported that the amount of between-study variability in results was a reason not to perform a meta-analysis. Other additional reasons given for not performing a meta-analysis were low quality of the primary studies (*n*=4), too few primary studies (*n*=2), and studies having different cut-offs for defining a positive test result (*n*=1).

When obtaining summary estimates of test accuracy, about a third of the reviews used a more advanced hierarchical bivariate random effects model: 13 (25 %) used a bivariate random effects model and 5 (9 %) used a hierarchical summary ROC (HSROC) model [9, 10]. Almost half used the Moses-Littenberg summary ROC approach (*n*=24; 45 %) [8]. A quarter of the meta-analyses only used a univariate approach, pooling results for the summary estimates separately (*n*=13; 25 %).

### Visual presentation of variability

The various approaches and measures to investigate and report variability in results are summarized in Table 2. The first step in assessing variability is typically to examine the study results visually, either through a forest plot per accuracy measure or by plotting pairs of sensitivity and specificity together in ROC space. Thirty-four of the 53 reviews with a meta-analysis (64 %) contained forest plots for at least one accuracy measure. Even more presented results in an ROC plot (*n*=40; 75 %). Of these, approximately two-thirds (*n*=27) indicated the relative size of the studies by varying the size of the points. Three reviews went further to indicate the relative size of the diseased and non-diseased group by varying the vertical and horizontal size of the points. Five reviews (9 %) contained neither a forest nor a ROC plot.

**Table 2:** Methods for presenting, testing, measuring, and communicating variability in results (*n*=53)

Method	Number	Percent
Graphical		
Plots present in article		
No plots	5	9 %
Forest Plots	34	64 %
ROC	40	75 %
Both	26	49 %
Statistical		
Cochran’s Q test	28	53 %
I^2^	31	58 %
Confidence Intervals presented	7	13 %
τ^2^	7	13 %
From a univariate analysis	6	11 %
From a bivariate analysis	1	2 %
Prediction intervals, ellipses, or bands	3	6 %
Provided a definition for significant variability	24	49 %
Cochran’s Q test	10	19 %
I^2^	7	11 %
Cochran’s Q test or I^2^	7	13 %
Influence of variability on analysis approach (reported by authors)		
Whether to perform a meta-analysis in the first place^a^	1^a^	4 %^a^
Whether to use a fixed or a random effects model	16	30 %
Whether to investigate sources of heterogeneity	4	8 %
How variability in results is mentioned in the abstract and discussion and/or conclusions	Discussion/Conclusions	Abstract
Any mention of variability listed below	29 (55 %)	15 (28 %)
A vague discussion variability^b^	17 (32 %)	10 (19 %)
Reported results of a statistical test or measurement of variability	N/A	4 (8 %)
Variability in results precludes firm conclusions or is a study limitation	13 (25 %)	2 (4 %)
Despite variability in results, a conclusion could still be made	7 (13 %)	3 (6 %)
There was no relevant variability in results	1 (2 %)	2 (4 %)

^a^ The denominator for this result is the 12 systematic reviews which did not contain a meta-analysis

^b^ Ex.: “sensitivities of studies varied widely”

### Testing for and quantifying statistical heterogeneity

The methods used for testing and quantifying heterogeneity per parameter can be found in Table 3. Cochran’s Q test for statistical heterogeneity was used in about half of the reviews (*n*=28; 53 %). The same was true for the inconsistency index (I^2^) (*n*=31; 58 %). The confidence interval for I^2^ was only provided in 7 of these 31 reviews. Cochran’s Q test and I^2^ were only used for univariate analyses, in other words, only on one accuracy measure at a time. Some reviews (also) used these metrics on an “overall measure” for accuracy such as the DOR (Cochran’s Q test, *n*=9; I^2^, *n*=10) or percentage total agreement (Cochran’s Q test, *n*=2; I^2^, *n*=2). Other reviews used at least one of these metrics on likelihood ratios (*n*=9) and/or predictive values (*n*=3).

**Table 3:** Measures of statistical heterogeneity per type of accuracy estimator (*n*=53)

	Cochran’s Q test n(%)	I^2^ n(%)	τ^2^ n(%)	Any test or measurement n(%)
Sensitivity and/or specificity	22 (42 %)	24 (45 %)	4 (8 %)	31 (58 %)
Predictive values	3 (6 %)	3 (6 %)	1 (2 %)	4 (8 %)
DOR	9 (17 %)	10 (19 %)	1 (2 %)	13 (25 %)
Accuracy	2 (4 %)	2 (4 %)	1 (2 %)	3 (6 %)
Likelihood Ratio	9 (17 %)	5 (9 %)	3 (6 %)	10 (19 %)
Any parameter	31 (58 %)	26 (49 %)	7 (13 %)	

About half of the articles described how they would consider whether variation beyond chance was present or relevant (*n*=24; 49 %). Of these, 10 based their conclusion on the p-value from Cochran’s Q test, 7 looked at the I^2,^ and the remaining 7 relied on both. Reviews were classified as having used both statistics simultaneously when it was unclear which one was used to draw conclusions. For example, one study reported, “Heterogeneity was assessed using the Q and I^2^ tests. The results were considered to be significant when *p*<0.1 or I^2^> 50 %.” [14].

Of the 10 reviews which derived their conclusions only from Cochran’s Q test, 8 considered there to be statistical heterogeneity when the p-value was <0.05 while the other 2 chose a less strict p-value of <0.1. For the 7 reviews which based their conclusions only on I^2^, 4 provided ranges for what was considered low, medium, or high variability. The ranges provided were different amongst these 4 reviews. Three meta-analyses only mentioned one cut-off for I^2^; if I^2^ was >50 %, they considered there to be statistical heterogeneity. Of the 7 reviews that relied on both Cochran’s Q test and the I^2^, all used the cutoff of >50 % for I^2^, and for the p-value for the Q test; 4 used <.05 and 3 used <0.1.

The between-study variance, τ^2^, which comes from a random effects model, was reported in 7 reviews (13 %). In 6 of these reviews, the τ^2^ was from a univariate analysis (in these studies no bivariate analysis was performed), and in one study the τ^2^s came from a bivariate random effects model. Prediction regions were only reported in 3 reviews (6 %), of which 1 study reported prediction intervals for sensitivity and specificity separately and 2 drew prediction ellipses in ROC space.

Threshold effects were assessed as a source of variability in 20 meta-analyses; 15 looked into implicit variations of the index test (e.g., a higher vs. lower resolution imaging test) and 7 investigated explicit differences (e.g., different cut-off points).

### Influence of variability on analysis decisions

Authors reported that the results of investigation of variability would guide choices in the analysis. Sixteen (32 %) said that they would use a random effects model if there was high variability, but otherwise use a fixed effect model. Ten of these 16 provided a definition for high variability. Four (8 %) said that they would only investigate sources of variability if there was high variability, of which all but one defined high variability.

### Incorporation of the results about variability in concluding remarks

Differences were found in the importance placed on the results of the quantification of variability. We considered articles reporting on the magnitude of the variability in estimates either in the abstract or in the discussion and/or conclusion section (hereafter referred to as the conclusions) to have put a high importance on it. More than half of the reviews mentioned something about it in the conclusions section (*n*=29; 55 %), while about a third mentioned it in the abstract (*n*=15; 28 %).

Several articles vaguely addressed the amount of variability (e.g., “the sensitivity in studies varied widely”), 17 in the conclusions (32 %) and 10 in the abstract (19 %). Four reviews (8 %) reported a variability measure in the abstract. A relatively large proportion mentioned that high variability precluded them from making firm conclusions or reported that it was a study limitation: 13 in the conclusions (25 %) and 2 also in the abstract (4 %). On the other hand, a few reviews mentioned that despite the variability in results, they were still able to make conclusions: 7 in the conclusions (13 %) and 3 also in the abstract (6 %). Two reviews reported (in either the conclusions or the abstract) that their analysis revealed no relevant variability in results.

## Discussion

### Key findings of this review

We found that more than half of the meta-analyses of diagnostic accuracy studies tested for statistical heterogeneity using Cochran’s Q test (*n*=28, 53 %) or quantified it using I^2^ (*n*=31, 58 %). They did this for univariate accuracy measures: either sensitivity and specificity separately, or the DOR. Although the DOR may be more homogenous across studies because opposite changes in sensitivity and specificity may get cancelled out, it is critical to detect and report these opposite changes when evaluating the clinical use of a test as the consequences of false-positive findings (specificity) and false negative findings (sensitivity) are hardly ever the same. Only about one third (*n*=18, 34 %) of the meta-analyses performed a bivariate random effects model. Of these reviews, only 1 reported τ^2^, and only 2 reviews drew prediction ellipses in ROC space.

While most reviews made use of univariate statistics, such as Cochran’s Q test (chi-square test) and I^2^, the use of these statistics should be at least be questioned, if not discouraged, as they cannot separate out variability due to the threshold effect [3]. A bivariate analog to the I^2^ has recently been developed, however research and guidance on its use in reviews on diagnostic tests is needed [15, 16]. The recommended hierarchal bivariate models provide insight into variability beyond that which can be explained by the threshold effect. In particular, the bivariate random effects model enables the calculation of the conditional between-study variances (i.e., the variance in specificity at a fixed value of sensitivity and vice versa). When there is a threshold effect (i.e., correlation), the conditional between-study variances will be smaller than the between-study variances from two separate univariate analyses of sensitivity and specificity.

Only about one third of the meta-analyses in this review performed the widely recommended analysis method of choice, a bivariate random effects model or the hierarchical summary ROC approach [3, 11]. There are situations when it is not possible to use such a model, such as when there are very few primary studies, as well as situations in which researchers may only be interested in one accuracy measure. However, the low percentage of meta-analyses using a hierarchical bivariate random effects model argues for the need for more guidance on the implementation and interpretation of such models.

There is room for improvement in the way that variability is quantified and reported in meta-analyses of diagnostic studies. Although a large portion of the quantification of variability is currently subjective because methods for quantification in reviews of studies on diagnostic test accuracy are under development, defining what one considers to be a significant amount of variability and reporting the results of the quantification of variability enhances the readers the ability to interpret the results. Review authors should also offer explanations of what these metrics mean and what the practical implications may be of the amount of variability observed.

### Comparison with other literature

As the field of meta-analyses is rapidly developing, it is difficult to compare our findings directly to those from existing meta-epidemiological research on reviews of diagnostic studies [1, 13]. The I^2^ was introduced in 2002 and the bivariate random effects model for sensitivity and specificity was introduced in 2005 [9]. Compared to prior reviews, our review found that the use of Cochran’s Q test remains high and that the I^2^ is now also commonly used alongside it. While the use of bivariate random effects meta-analyses is increasing [2], our review found that the between study variances (τ^2^s) that are estimated from this approach are not always reported and the prediction regions are rarely reported.

### Strengths and weaknesses of this review

While our review contains a relatively small set of systematic reviews published within a short timeframe, we do not think that this is a major weakness. As there have been many advances in reviews on diagnostic tests since the existing reviews on this topic were performed, our review, which contains a recent set of publications, is still highly relevant for current practice. Additionally, while our search strategy could have missed some relevant reviews, we think that the representative sample of over 50 reviews was enough to obtain theoretical saturation. In other words, that including more reviews would not have significantly changed our conclusions [17]. A limitation of this review, which is true for any review on methodology, is that we observed only what authors reported having done, not what they actually did.

### Initial guidance

**Fig. 1:**
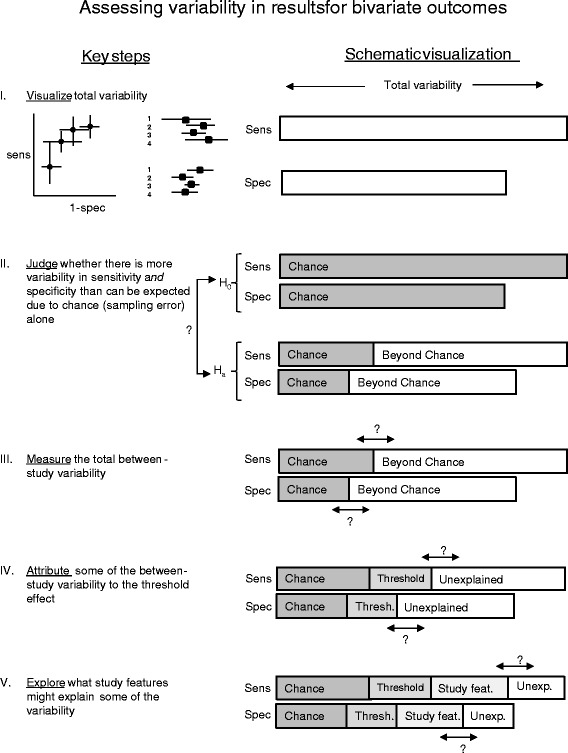
Steps for assessing variability in reviews of diagnostic tests when there are two potentially correlated outcomes of interest, sensitivity and specificity

While a high level of importance was generally given to the quantification of variability, as can be seen by the high frequency with which it was mentioned in the prominent sections of the paper, there is room for improvement. There is a need for formal guidance on the quantification of variability in meta-analyses of diagnostic studies. Below we provide an outline of what such guidance may look like, focusing on the bivariate random effects model framework (Fig. 1).

I. Visualize total variability in sensitivity and specificity by presenting the data in forest and ROC plots, preferably showing the precision of the estimates using crosshairs or by varying the size of the points.
II. Judge whether there is more variability in sensitivity and specificity than could be expected due to chance alone through visual inspection of the data. While Cochran’s Q test facilitates this judgment in meta-analyses of univariate outcomes, no such analogue has been developed for the bivariate diagnostic setting to our knowledge. Such test(s) could detect beyond-chance variability around a summary point or around a curve in ROC space. For now, visualizing results in ROC space may help determine which hierarchical bivariate random effects model to use (the bivariate random effects model which focuses on estimating a summary point or the HSROC which focuses on estimating a summary curve). Additionally, if the hierarchical bivariate model fails to converge or provides unreliable estimates (as is often the case when there are few studies or sparse data due to extreme sensitivity or specificity), observation of the data on the plot can guide decisions on how to simplify the analyses [18].
III. Measure total between-study variability in sensitivity and in specificity (τ^2^s) by fitting a (bivariate) random effects model. Interpretation of the τ^2^s can be facilitated by presenting confidence and prediction intervals [19]. If a bivariate random effects model fails, two separate univariate random effects models should be considered. The τ^2^s from these univariate random effects models express the amount of variability that cannot be explained by chance, assuming no correlation between sensitivity and specificity. In univariate analyses, I^2^ provides a measure of variability. While an analog has been developed for the bivariate setting, more research and guidance is needed on how to implement it reviews on diagnostic tests is needed [15, 16].
IV. Attribute some of the between study-variability to the threshold effect by fitting a bivariate random effects model. The conditional between-study variability for sensitivity and for specificity (the conditional τ^2^s) is the variability beyond both chance and the threshold effect. It can be calculated using the covariance and the total between-study variances. Both confidence and prediction ellipses should be provided to assist interpretation.
V. Explore what might be causing the systematic differences in accuracy estimates between the primary studies [20]. Again, a hierarchical bivariate random effects model is a sound and flexible approach for investigating the impact of study characteristics on sensitivity or specificity or both.

The quantification of variability is not simply a methodological exercise; variability in results can have implications for clinical practice. While consistency of results across studies strengthens overall findings, inconsistency can complicate interpretations. The more variability beyond chance that has not been explained, the more difficult it is to come to strong conclusions about the clinical implications of the findings of the meta-analysis [7]. Investigations of variability identify important differences in test performance across patient subgroups, variations of the test(s) under study, or study designs. The more variability that can be explained by sample size, threshold effects, or study characteristics, the more confidence can be placed in the results.

Ideally, variability in results can best be addressed in individual participant data meta-analyses. Unfortunately, however, there are many barriers to accessing and combining the primary data to perform such analyses.

## Conclusion

In conclusion, approaches to assessing variability in estimates of accuracy varied widely between diagnostic test accuracy reviews and there is room for improvement. We have provided initial guidance in this area, complemented by an overview of the currently available approaches.

## Additional files

Additional file 1:
**Search Strategy.** (DOCX 14 kb) Additional file 2:
**Overview of methods for investigating variability in univariate meta-analysis [** 5, 6, 19–24 **].** **Figure S1.** Overview of methods and measure for assessing variability in univariate outcomes. (DOCX 93 kb) Additional file 3:
**Study inclusion flow chart.** (DOCX 96 kb) Additional file 4:
**List of articles included in the review.** (DOCX 21 kb)

## Abbreviations

DOR: diagnostic odds ratio
HSROC: hierarchical SROC
SROC: summary receiver operating characteristic curve
