# Supplementary material for: Assessing variability in results in systematic reviews of diagnostic studies
Source: BMC Med Res Methodol. 2016 Jan 15;16:6. doi: 10.1186/s12874-016-0108-4 (PMC4714528; doi:10.1186/s12874-016-0108-4)
Supplement: Additional file 1: — Search Strategy. (DOCX 14 kb) [file 12874_2016_108_MOESM1_ESM.docx]

Additional file 1. Search Strategy

1. systematic.mp. [mp=ti, ab, sh, hw, tn, ot, dm, mf, dv, kw]

2. limit 1 to "reviews (best balance of sensitivity and specificity)"

3. predict*.ti,ab.

4. test.ti,ab.

5. tests.ti,ab.

6. 4 or 5

7. 2 and 3 and 6

8. screen*.mp. [mp=ti, ab, sh, hw, tn, ot, dm, mf, dv, kw]

9. 2 and 8

10. monitoring.mp. [mp=ti, ab, sh, hw, tn, ot, dm, mf, dv, kw]

11. 2 and 10

12. "multiple tests".mp. [mp=ti, ab, sh, hw, tn, ot, dm, mf, dv, kw]

13. 2 and 12

14. "diagnostic test accuracy".mp. [mp=ti, ab, sh, hw, tn, ot, dm, mf, dv, kw]

15. DTA.ti,ab.

16. exp "sensitivity and specificity"/

17. specificit*.tw.

18. "false negative".tw.

19. accuracy.tw.

20. 14 or 15 or 16 or 17 or 18 or 19

21. 2 and 20

22. 7 or 9 or 11 or 13 or 21

23. limit 22 to (english language and yr="2011 -2013")
