# Supplementary material for: Assessing variability in results in systematic reviews of diagnostic studies
Source: BMC Med Res Methodol. 2016 Jan 15;16:6. doi: 10.1186/s12874-016-0108-4 (PMC4714528; doi:10.1186/s12874-016-0108-4)
Supplement: Additional file 2: — Overview of methods for investigating variability in univariate meta-analysis [ 5, 6, 19–24 ]. Figure S1. Overview of methods and measure for assessing variability in univariate outcomes. (DOCX 93 kb) [file 12874_2016_108_MOESM2_ESM.docx]

**Additional file 2. Overview of methods for investigating variability in *univariate* meta-analysis**

Investigating variability in results across studies in a meta-analysis of a *univaraite* outcome involves four steps: 1) visualizing the total variability 2) asking whether there more variability in results than can be expected due to chance. 3) If so, measuring how large the variability beyond chance is and 4) exploring what study factors might be able to explain some of this variability beyond chance? We highlight how the questions on variability in results mentioned at the beginning of this section above can be examined from a univariate perspective in Figure A. Please that the purpose of this is to provide an overview of the methods used with univariate outcomes, not to recommend them for use with bivariate outcomes.

*Statistical Heterogeneity*

The goal of the first question, “Is there more variability in results than can be expected due to chance alone?”, is to determine whether the “true effect” measures vary between studies, or in other words, whether there is statistical heterogeneity. Cochran’s Q test can be used to test whether chance alone could explain the variation in univariate outcomes.[[21](#_ENREF_21), [22](#_ENREF_22)] It is worth noting that this statistic is dependent upon both the number and the size of the studies in a meta-analysis.[[23](#_ENREF_23)] When there are a small number of studies, the test is underpowered, meaning that it will fail to detect true heterogeneity. When more variability in estimates than can be attributed to chance alone is likely, random effects models are preferable.

*Quantifying between-study variability*

When there is more variability than can be attributed to chance alone, it is relevant ask “How large is the variability in “true effects between studies?” A popular metric for statistical heterogeneity is the inconsistency index, I^2^, which is defined as the percentage of the total variability in results that cannot be attributed to chance.[[6](#_ENREF_6), [24](#_ENREF_24)]

Another metric for variability is the between-study variance estimated from the random effects model, τ^2^.[[5](#_ENREF_5), [19](#_ENREF_19)] This is an estimate of the variance of the true underlying effects from the individual studies around the average effect. Prediction intervals can also be calculated using the results from a random effects model. A prediction interval is the region in which the “true effect” measure (i.e. estimate from a large study) of a new, comparable study is expected to be found.

*Exploring Sources of Variability*

When it is concluded that there is a sizeable amount of variability than cannot be attributed to chance, it is relevant to ask, “What study characteristics might be able to explain some of this variability beyond chance?” Differences in the distribution of clinical factors, such as the age or disease severity of the patients, or methodological factors, such as whether blinding occurred, may be causing different accuracy measures across studies. It is relevant to know, for example, if a test performs better in some patients than others, or if different types of studies (for example those with a high risk of bias) show different results.

Many approaches exist for exploring sources of variability.[[20](#_ENREF_20)] Potential sources of variability can be explored through sensitivity analysis, stratified analysis, or meta-regression. In sensitivity and stratified analyses, variability in the remaining studies or new subgroups can be quantified. Remaining variability will be lower, if subgroups differ (on average) in their estimates. If there is significantly less variability in the subgroups, then some of the variation in results may be attributed to the defining factor of the subgroup(s). In meta-regression, factors thought to be possible sources of variability are added as covariates to the model. In random effects meta-regression, the τ^2^s of the new model represent the remaining, still unexplained variability.


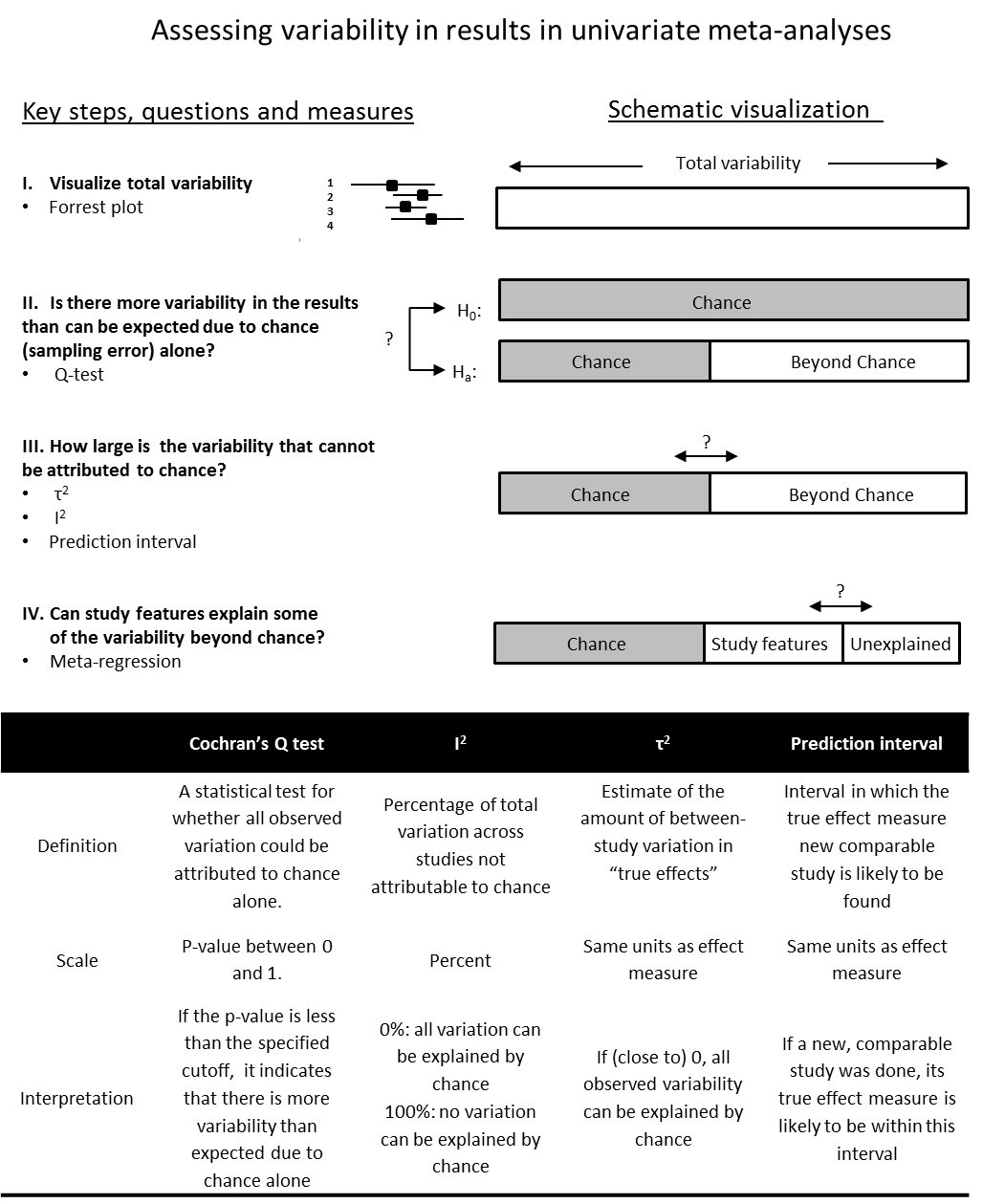


Figure S1. Overview of methods and measure for assessing variability in univariate outcomes
