# Supplementary figures and images for: Assessing variability in results in systematic reviews of diagnostic studies
Source: BMC Med Res Methodol. 2016 Jan 15;16:6. doi: 10.1186/s12874-016-0108-4 (PMC4714528; doi:10.1186/s12874-016-0108-4)

Additional file 3. Study inclusion flow chart


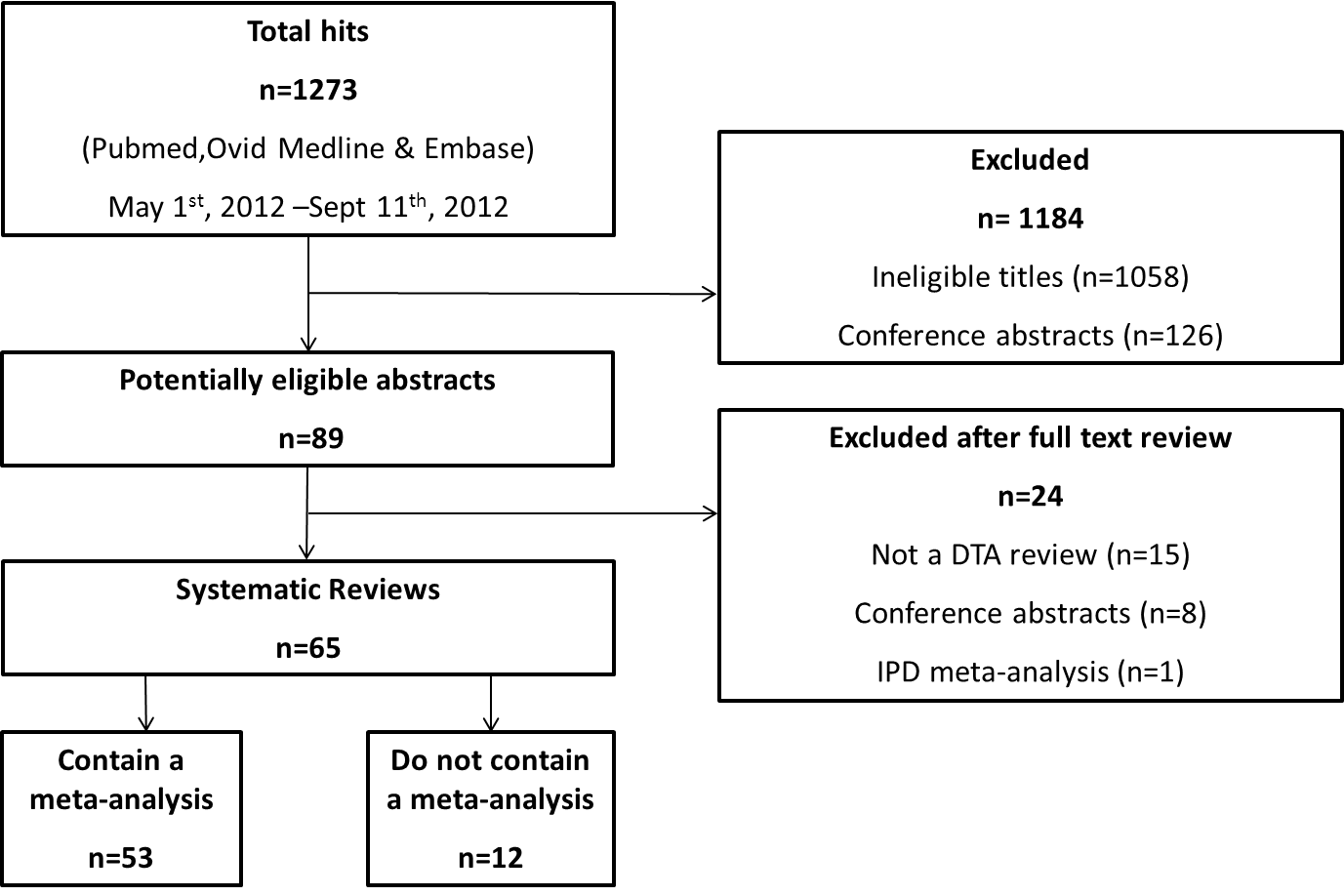

Supplement: Additional file 3: — Study inclusion flow chart. (DOCX 96 kb) [file 12874_2016_108_MOESM3_ESM.docx]
